# Case Report: Anti-NMDAR encephalitis associated with neurobrucellosis: causality or coexistence?

**DOI:** 10.3389/fimmu.2025.1536740

**Published:** 2025-05-15

**Authors:** Yao Wang, Xue Ma, Chao Ma, Tangna Sun, Daidi Zhao, Hongzeng Li, Yaping Yan, Jun Guo

**Affiliations:** ^1^ Department of Neurology, Tangdu Hospital, The Fourth Military Medical University, Xi’an, China; ^2^ Department of Neurology, The First Affiliated Hospital of Xi’an Jiao Tong University, Xi’an, China; ^3^ Department of Cardiology, Tangdu Hospital, The Fourth Military Medical University, Xi’an, China; ^4^ College of Life Sciences, Shaanxi Normal University, Xi’an, China

**Keywords:** neurobrucellosis, anti-NMDAR antibodies, encephalitis, immunotherapy, causality

## Abstract

Human brucellosis, caused by *Brucella*, is an infectious disease with specific endemic regions, especially in pastoral areas, and may affect multiple organ systems. Neurological involvement, namely neurobrucellosis, occurs in very few of these patients. Anti-N-methyl-D-aspartate receptor (NMDAR) encephalitis is the most frequent type of autoimmune encephalitis and is usually associated with tumors or herpes simplex virus infections. However, the link between the two disease entities is unknown. In this report, we present a rare case of a 29-year-old Chinese man with anti-NMDAR encephalitis associated with neurobrucellosis, with the detection of anti-NMDAR antibodies by cell-based assay and *Brucella melitensis* by metagenomic next-generation sequencing in his cerebrospinal fluid sample. The patient improved after antimicrobial treatment and immunotherapies, including steroids and intravenous immunoglobulin. This case implicates *Brucella* infection as a possible trigger for the production of anti-NMDAR antibodies, and prospective studies should reveal whether there is a casual relationship between brucellosis and anti-NMDAR antibodies.

## 1 Introduction

Human brucellosis caused by the bacterial genus *Brucella melitensis* involves multiple organs with a broad spectrum of clinical manifestations through direct or indirect contact with animals or the inhalation of infected aerosolized particles (1). Clinical manifestations of brucellosis include fever, malaise, arthralgias, hepatosplenomegaly, lymphadenopathy, and several complications, such as osteoarticular diseases, epididymoorchitis, and central nervous system (CNS) disorders (1). CNS involvement in brucellosis, known as neurobrucellosis, is a rare and heterogeneous disease that has no distinctive clinical presentations (2). Meningitis, meningoencephalitis, encephalitis, cerebrovascular diseases, brain abscesses, demyelinating syndromes, and myelopathy have all been reported (3, 4).

Anti-N-methyl-D-aspartate receptor (NMDAR) encephalitis is an autoimmune disease clinically characterized by neuropsychiatric disturbances, memory problems, and the detection of antibodies against NMDAR (5). The known common triggers of anti-NMDAR encephalitis are tumors (6, 7) or herpes simplex virus infections (8, 9). A case of *Brucella* infection complicated by anti-NMDAR encephalitis has been documented (10). A recent study hypothesized that *Brucella* infection may precipitate psychotic symptoms analogous to those observed in anti-NMDAR encephalitis (11). Notably, neurobrucellosis complicated by anti-NMDAR encephalitis remains an extremely rare clinical entity, with limited case reports documented in the literature. In this article, we present a rare case of a young Chinese man with anti-NMDAR encephalitis secondary to neurobrucellosis.

## 2 Case presentation

**Figure 1:**
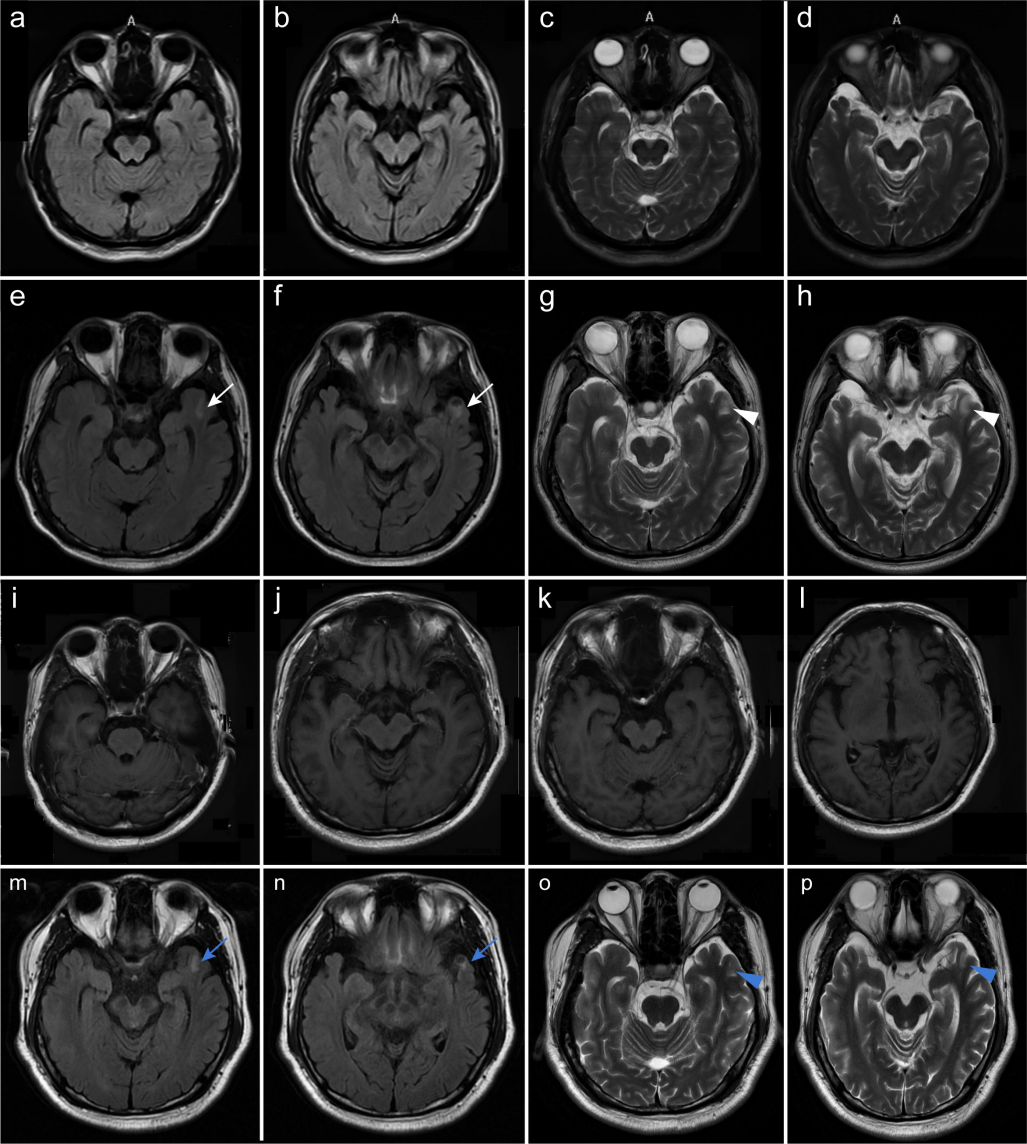
Brain MRI of the patient. Axial brain FLAIR **(a, b, e, f, i, j)** and T2-weighted images **(c, d, g, h, k, l)** are shown. No abnormalities were observed on brain MRI images **(a–d)** of the patient at his initial admission to our department. The patient developed a new and ill-defined patchy lesion in the left temporal lobe [**(e–h)**; white arrows] nearly 1 month later. No significant enhancement was observed on contrast-enhanced MRI **(i–l)**. Following 6 months of immunotherapies including plasma exchange, IVIg, and steroids, the lesion was confirmed to have shrunk [**(m–p)**; blue arrows].

In June 2023, a 29-year-old Chinese man presenting with a 1-month headache, diplopia, and occasional visual hallucinations was admitted to our hospital. He reported one episode of generalized tonic-clonic seizures lasting 2–3 minutes 7 months previously. He also complained of intermittent low-grade fever (37.8°C–38.0°C) of unknown origin accompanied by muscle and joint pain in the previous year. He had a history of contact with goats previously. His neurological examination on admission revealed limited abduction in both eyes and neck stiffness. The clinical findings of the patient were shown in **Table 1**. The modified Rankin Scale (mRS) score was 0. The results of routine laboratory tests, including blood cell assay, C reactive protein, erythrocyte sedimentation rate, and inflammatory cytokines, were all unremarkable. HBs-Ag, HCV-Ab, HIV test, and treponema pallidum antibodies were all negative. The rheumatological workup and neoplasm marker screening yielded normal results. Video-electroencephalography displayed a small amount of low-medium amplitude slow-wave activity during the awake period. No abnormalities were observed on brain magnetic resonance imaging (MRI) (**Figures 1a–d**). A lumbar puncture indicated an elevated intracranial pressure (ICP) (> 400 mmH_2_O; normal range 80–180 mmH_2_O). The cerebrospinal fluid (CSF) test showed moderate pleocytosis (168×10^6^/L; normal range<5×10^6^/L) with a percentage of mononuclear cells of 80%, decreased glucose (1.66 mmol/L; normal range 2.5–4.5 mmol/L), and an elevated protein level (1.32 g/L; normal range 0.15–0.45 g/L). The CSF IL-6 level was 338 pg/mL. *Brucella melitensis* was detected in the CSF by metagenomic next-generation sequencing (mNGS) with a specific read number of 204. Meanwhile, *Brucella* was grown in the CSF culture. Antibodies against *Brucella* were detected in the serum using the Rose Bengal plate test. Both CSF and serum samples were tested by cell-based assay or immunodot assays for paraneoplastic antibodies, including anti-Hu, anti-Yo, anti-Ri, anti-Ma2, anti-CV2, and anti-amphiphysin, and for autoantibodies associated with CNS inflammatory demyelinating diseases and autoimmune encephalitis, including anti-AQP4, anti-MOG, anti-GFAP, anti-NMDAR, anti-AMPAR, anti-LGI1, anti-GABA_B_R, anti-CASPR2, anti-DPPX, anti-IgLON5, anti-mGluR5, anti-neurexin-3α, and anti-GAD65. Among them, only the anti-NMDAR antibodies were positive with a low titer of 1:10, but there was a negative result in the subsequent tissue-based assays (TBA).

**Table 1:** Clinical findings of the patient before and after antimicrobial therapy.

Items	Before treatment	After treatment				
		9 days	27 days	59 days	153 days	277 days
mRS score	0	0	5	2	1	0
Intracranial pressure (mmH_2_O)	> 400	210	280	160	170	170
CSF protein level (g/L)	1.32	1.27	0.90	0.40	0.36	0.28
CSF WBC count (×10^6^/L)	168	78	36	16	2	0
CSF glucose level (mmol/L)	1.66	1.42	2.43	3.42	3.91	3.86
CSF IL-6 level (pg/mL)	338	454	35.1	5.08	4.94	4.81
CSF *Brucella melitensis* read number	204	NA	85	13	0	0
Serum anti-NMDAR antibodies titer	1:10	NA	1:100	Negative	Negative	Negative
CSF anti-NMDAR antibodies titer	1:10	NA	1:100	1:3.2	1:3.2	1:1
Brain MRI	Normal	NA	A new-onset lesion in the left temporal lobe	NA	NA	The lesion in the left temporal lobe shrunk

NA, not available; CSF, cerebrospinal fluid; NMDAR, N-methyl-D-aspartate receptor; WBC, white blood cell; IL-6, interleukin-6; MRI, magnetic resonance imaging; mRS, modified Rankin Scale.

**Figure 2:**
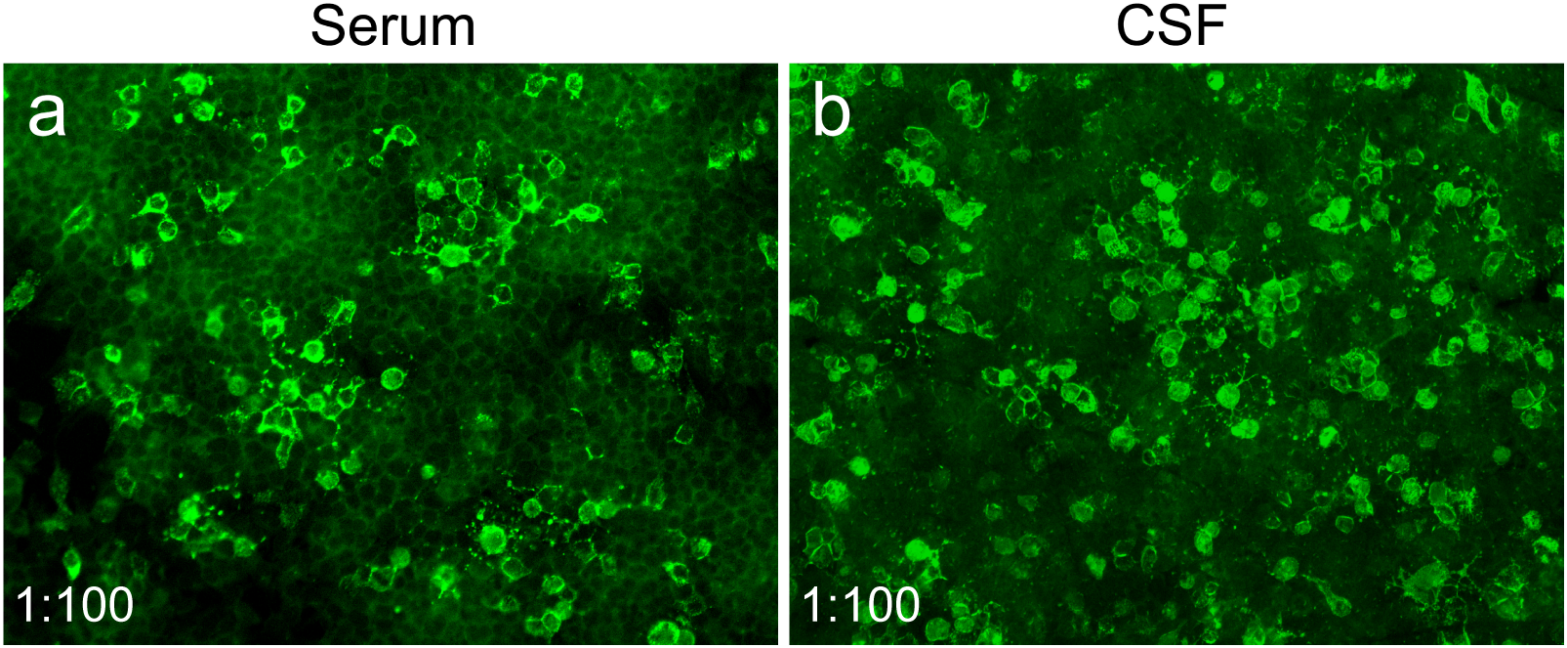
Serum and CSF anti-NMDAR antibodies of the patient were detected by cell-based assay. Representative images of positive serum **(a)** and CSF **(b)** anti-NMDAR antibodies at 27 days after antimicrobial therapy (1:100 in both). Indirect immunofluorescence staining was performed using Alexa Fluor 488-conjugated secondary antibodies.

Identifying *Brucella melitensis* in the CSF led to a definite diagnosis of neurobrucellosis. His intracranial hypertension-related symptoms, including headache and diplopia, rapidly resolved following treatment with first-line antimicrobial regimens, including ceftriaxone (2 g twice daily), doxycycline hydrochloride (100 mg twice daily), rifampin (0.6 g once daily), and mannitol. After 9 days, the patient’s ICP declined to 210 mmH_2_O, accompanied by decreases in CSF white blood cell (WBC) count (78×10^6^/L) and protein level (1.27 g/L), despite low CSF glucose (1.42 mmol/L) and increased IL-6 levels (454 pg/mL). The patient then continued to receive this treatment regimen and was discharged from our hospital. However, his body temperature reached 38.6°C 10 days later, and the local clinicians prescribed a second-line regimen with compound sulfamethoxazole (800 mg twice daily) instead of ceftriaxone. Unfortunately, he developed psychosis, aggressive behaviors, and kidney failure approximately 6 days later. Repeat mNGS analysis showed that the number of *Brucella melitensis* reads in the CSF decreased to 85. The WBC count in the CSF decreased to 36×10^6^/L and the protein level to 0.90 g/L, and CSF glucose increased to 2.43 mmol/L. The concomitant CSF IL-6 level was 35.1 pg/mL. Unexpectedly, elevated titers of CSF and serum anti-NMDAR antibodies were identified by cell-based assay (1:100 in both; **Figure 2**), and a positive staining was confirmed in the following TBA, thus supporting the development of anti-NMDAR encephalitis, with an mRS score of 5. Meanwhile, a repeat brain MRI showed a new-onset lesion in the left temporal lobe on fluid attenuated inversion recovery (FLAIR) (**Figures 1e, f**) and T2-weighted images (**Figures 1g, h**). No significant enhancement was observed on contrast-enhanced MRI (**Figures 1m–p**). His renal function improved following continuous renal replacement therapy (CRRT) for 3 days and fluid supplementation. Considering the potential exacerbation of brucellosis by high-dose glucocorticoids, plasma exchange followed by intravenous immunoglobulin (IVIg) combined with low-dose dexamethasone was given. The patient’s mania and aggressive behaviors ameliorated gradually with 1-month immunotherapy, with an mRS score of 2. Repeat CSF analysis showed reduced WBC count, protein level, and *Brucella*-specific read number (16×10^6^/L; 0.40 g/L; 13). The CSF anti-NMDAR antibodies titer declined to 1:3.2, with a negative result in serum.

Subsequently, combination therapy of oral prednisone at 40 mg per day with a tapering schedule of 5 mg every week and antimicrobial drugs, including moxifloxacin (0.4 g once daily), doxycycline hydrochloride (100 mg twice daily), and rifampin (0.6 g once daily), was administered. After 3 months, he still suffered from mild neuropsychiatric symptoms, manifesting as visual hallucinations and euphoria. *Brucella* was not detected in CSF by mNGS. However, the CSF anti-NMDAR antibodies titer remained at 1:3.2. Thus, his antimicrobial treatment was discontinued, and he received intravenous methylprednisolone (500 mg per day for 5 days), followed by oral prednisolone (25 mg once daily) with a tapering of 5 mg every week. His psychiatric disturbances disappeared and CSF results returned to normal 3 months later. Brain MRI confirmed that the lesion in the left temporal lobe had shrunk dramatically (**Figures 1i–l**). In the outpatient follow-up in March 2024, the patient remained stable in clinical status, and the mRS score declined to 0. Up to the recent follow-up by telephone in July 2024, he still reported no complaints.

## 3 Discussion

Human brucellosis caused by *Brucella melitensis* shows a myriad of clinical manifestations attributed to the involvement of various organs, including cardiovascular, gastrointestinal, hepatobiliary, genitourinary, musculoskeletal, CNS, and peripheral nervous system (12). The incidence of neurological involvement in brucellosis is approximately 4% (1, 13). Neurological symptoms primarily comprise behavioral changes, meningitis, seizures, and cranial and peripheral neuropathies (14). Anti-NMDAR encephalitis is an immune-mediated disorder characterized by prominent neuropsychiatric symptoms and the presence of CFS anti-NMDAR antibodies (5, 15). The known triggers of NMDAR autoimmunity are tumors, usually ovarian teratoma, and herpes simplex encephalitis (5). The coexistence of neurobrucellosis with NMDAR autoimmunity is very rare.

**Figure 3:**
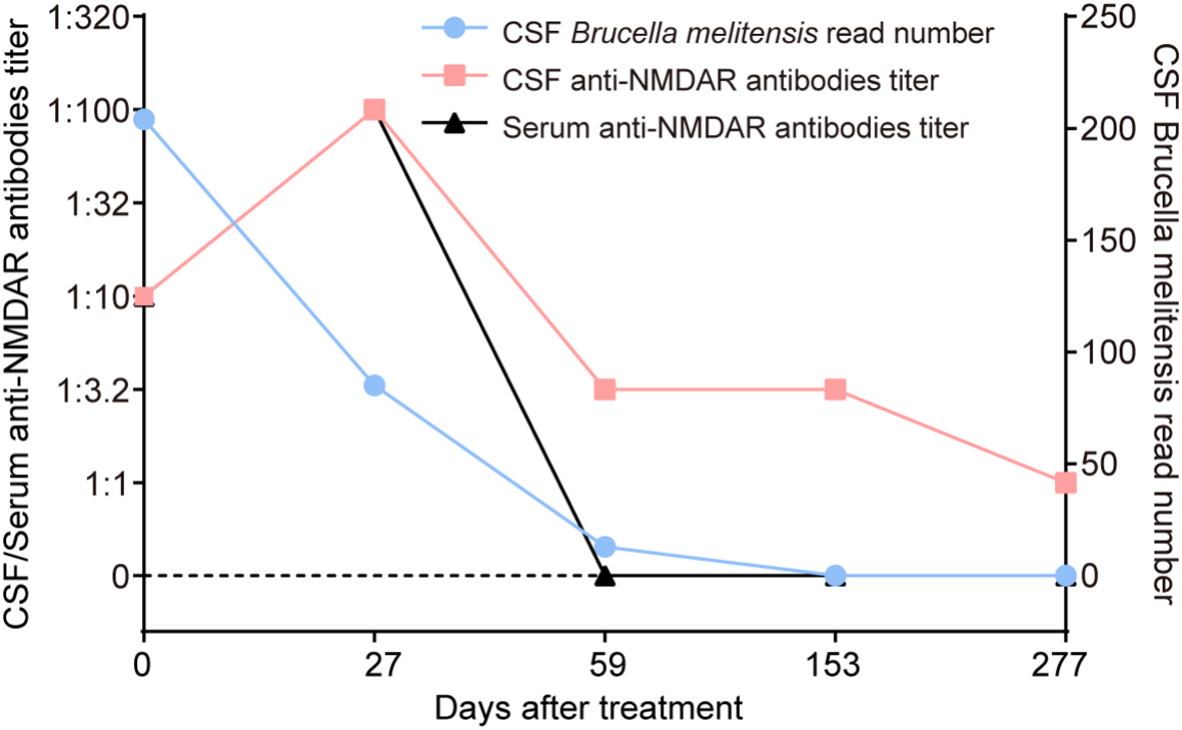
Changes in CSF *Brucella melitensis* read number, and serum and CSF anti-NMDAR antibodies of the patient. After 153 days of antimicrobial treatment, the CSF *Brucella melitensis* read number gradually decreased from 204 to 0. However, titers of serum and CSF anti-NMDAR antibodies dramatically increased from 1:10 to 1:100 at 27 days after antimicrobial therapy, which was accompanied by the occurrence of severe psychiatric symptoms. At the last follow-up, serum anti-NMDAR antibodies were negative, and the titer of CSF anti-NMDAR antibodies was reduced to 1:1.

Numerous studies have proven a potential association between infection and autoimmunity (16). For instance, a causal relationship between herpes simplex virus type 1 encephalitis and anti-NMDAR encephalitis was established (9, 17, 18). A previous study found that brucellosis might be a potential etiological factor for immunity dysregulation (3). In this case, both CSF and serum anti-NMDAR antibodies were positive at admission, but a lower antibody titer of 1:10 with negative results in TBA rendered the diagnosis of anti-NMDAR encephalitis uncertain, particularly in the absence of psychiatric disturbances. Unexpectedly, severe psychiatric manifestations emerged following effective antimicrobial treatment and were then reversed by immunotherapies (**Figure 3**). Meanwhile, increased CSF and serum anti-NMDAR antibodies titers (1:100 in both) and new-onset temporal lesions were observed (**Table 1**, **Figure 3**). All the findings support a definite diagnosis of anti-NMDAR encephalitis. It was speculated that neurobrucellosis may have been responsible for the development of anti-NMDAR encephalitis in our case, since the intermittent fever, implying the possible presence of underlying *Brucella* infection, had lasted for approximately 1 year before the initial admission. Recently, autoimmune encephalitis post-herpes simplex encephalitis was regarded as a new immunological pattern in autoantibody-mediated diseases (19). We presumed that there might be a similar paradigm in this case. Immunological mechanisms, including the production of cytokines or chemokines and innate and adaptive immunity dysfunction, may contribute to the secondary production of anti-NMDAR antibodies. Similar findings were described in three patients with autoimmune glial fibrillary acidic protein astrocytopathy (20), anti-NMDAR encephalitis secondary to brucellosis (10), and Guillain-Barre syndrome in association with brucellosis (21), respectively, in the available literature. These case reports supported *Brucella* infection as a possible trigger for neurological autoimmune disorders. The exact immune mechanisms underlying the observation between brucellosis and autoimmunity need to be further clarified. However, considering that anti-NMDAR antibodies and *Brucella melitensis* were detected in the CSF samples collected at the same time during the first admission, we cannot completely rule out the possibility that this patient developed neurobrucellosis and anti-NMDAR encephalitis simultaneously.

When neurobrucellosis coexists with anti-NMDAR encephalitis, clinicians face a unique therapeutic imperative requiring simultaneous implementation of antimicrobial agents and immunomodulatory therapies. These dual conditions present distinct clinical challenges that fundamentally differ from managing either disease in isolation, demanding meticulous coordination to address the complex interplay between persistent bacterial infection and autoimmune-mediated neuronal dysfunction. The concurrent administration of antibiotic regimens targeting neurobrucellosis and immunotherapy, including corticosteroids, intravenous immunoglobulins, and potentially rituximab, is critical, necessitating careful monitoring for both infection and neuroimmunology throughout the treatment course.

## 4 Conclusion

Overall, this single case report indicates a potential relationship between anti-NMADR encephalitis and neurobrucellosis. Further experimental study is necessary to prove this to be causal or a coincidence. Clinicians should be aware of the possibility of autoimmune encephalitis when encountering neurological deterioration in patients with neurobrucellosis receiving sufficient antimicrobial treatments.

## Data Availability

The raw data supporting the conclusions of this article will be made available by the authors, without undue reservation.
